# Antenatal risk factors for peanut allergy in children

**DOI:** 10.1186/1710-1492-7-17

**Published:** 2011-10-04

**Authors:** Karen E Binkley, Chad Leaver, Joel G Ray

**Affiliations:** 1 Division of Clinical Immunology and Allergy, Department of Medicine, St. Michael's Hospital University of Toronto, Toronto, Ontario, Canada; 2 Sunnybrook Health Sciences Centre, Department of Medicine, University of Toronto, Toronto, Ontario, Canada; 3 Institute for Clinical Evaluative Sciences, University of Toronto, Toronto, Ontario, Canada; 4 Department of Medicine, St. Michael's Hospital University of Toronto, Toronto, Ontario, Canada; 5 Department of Obstetrics and Gynecology, St. Michael's Hospital University of Toronto, Toronto, Ontario, Canada; 6 Department of Health Policy Management and Evaluation St. Michael's Hospital, University of Toronto, Toronto, Ontario, Canada

**Keywords:** Allergy, peanut, shellfish, prenatal, antenatal, pregnancy, folic acid, Rh immune globulin, survey

## Abstract

**Background:**

Prenatal factors may contribute to the development of peanut allergy. We evaluated the risk of childhood peanut allergy in association with pregnancy exposure to Rh immune globulin, folic acid and ingestion of peanut-containing foods.

**Methods:**

We conducted a web-based case-control survey using the Anaphylaxis Canada Registry, a pre-existing database of persons with a history of anaphylaxis. A total of 1300 case children with reported peanut allergy were compared to 113 control children with shellfish allergy. All were evaluated for maternal exposure in pregnancy to Rh immune globulin and folic acid tablet supplements, as well as maternal avoidance of dietary peanut intake in pregnancy.

**Results:**

Receipt of Rh immune globulin in pregnancy was not associated with a higher risk of peanut allergy (odds ratio [OR] 0.86, 95% confidence interval [CI] 0.51 to 1.45), nor was initiation of folic acid tablet supplements before or after conception (OR 0.53, 95% CI 0.19 to 1.48). Complete avoidance of peanut-containing products in pregnancy was associated with a non-significantly lower risk of peanut allergy (OR 0.53, 95% CI 0.27 to 1.03).

**Conclusion:**

The risk of childhood peanut allergy was not modified by the following common maternal exposures in pregnancy: Rh immune globulin, folic acid or peanut-containing foods.

**Clinical implications:**

Rh immune globulin, folic acid supplement use and peanut avoidance in pregnancy have yet to be proven to modulate the risk of childhood anaphylaxis to peanuts.

**Capsule Summary:**

Identification of prenatal factors that contribute to peanut allergy might allow for prevention of this life-threatening condition. This article explores the role of three such factors.

## Introduction

Prenatal and early life factors may contribute to the subsequent development of allergic conditions in childhood [1]. A better understanding and prevention of exposure to such factors could theoretically lead to the rational amelioration of some common and potentially life-threatening allergic conditions. In this study, we focused on three potentially important factors in the prenatal period: Rh immune globulin, folic acid supplements and ingestion of peanut-containing foods.

Rh immune globulin is a blood derived product with known immunomodulatory effects [2-4]. It is administered to Rh-negative mothers at 28 weeks' gestation to prevent alloimmunization to fetal Rh antigens, or within 72 hours of an obstetrical delivery or pregnancy termination. About 15% of Caucasian women, 4% to 8% of women of African ancestry and less than 1% of Asian women are Rh negative. Concerns about the use of Rh immune globulin were raised by members of a patient support group, who noted that several mothers of peanut allergic children had received Rh immune globulin during their pregnancy with the affected child.

Routine folic acid tablet supplementation in pregnancy became widespread in the early 1990s. This roughly correlated with the period during which peanut allergy and other allergic conditions became more prevalent [1]. Dietary methyl donors, including folic acid, can influence the expression of certain genes through DNA methylation, an epigenetic phenomenon [5]. Such mechanisms have been postulated to play a role in the development of allergic conditions [6]. Key genes involved in the regulation of immune and allergic responses, including Fox P3, are known to be affected by methylation [7].

Concern has also been raised about the risk of childhood peanut allergy following ingestion during pregnancy and lactation [8]. This has led some to recommend dietary avoidance of all peanut products during pregnancy and lactation [9]. However, recent studies have questioned this approach, since the incidence of peanut allergy may actually be higher following avoidance of peanut products [1].

We explored the risk of childhood peanut allergy in association with pregnancy exposure to Rh immune globulin, folic acid and peanut-containing foods using a large Canadian database of children with a history of anaphylaxis.

## Methods

A retrospective web-based case-control study was completed using the Anaphylaxis Canada Registry. This registry is a pre-existing database comprising approximately 8,000 anaphylactic patients registered from across Canada [[10], accessed 2008]. Members of Anaphylaxis Canada form a group of highly motivated individuals who are eager to participate in research on severe allergy in children. Response rates to previous research projects are typically over 85%.

All study participants were previously registered with Anaphylaxis Canada, and have granted permission to Anaphylaxis Canada to contact them periodically with web-based surveys. Our case group comprised parents or caregivers of a child with a peanut allergy. The active control group consisted of parents or caregivers of a child with a non-peanut allergy, specifically a shellfish allergy. Those with more than one child with an allergy were asked to respond to details in regards to their first liveborn child.

For the current study, an email was sent out by Anaphylaxis Canada to all 2007 survey respondents of the *AC Member Survey 2007*, all of whom previously stated that they were a parent/guardian of a child with a life-threatening allergy. Individuals were asked to respond to additional questions for the current study (Appendix A).

Approval was granted by the Research Ethics Review Board at Sunnybrook Health Sciences Centre, University of Toronto, Toronto, Ontario, Canada. All participant identifiers were removed from the dataset by Anaphylaxis Canada prior to its release to the investigators.

### Statistical analysis

The risk of each exposure variable in pregnancy was examined in association with case status of a peanut allergy vs. control status of a shellfish allergy, and expressed as an odds ratio (OR) and 95% confidence interval (CI).

## Results

A total of 1300 mothers of cases with peanut allergy and 113 mothers of controls with shellfish allergy completed the current survey.

There were 812 (56.0%) mothers who reported they were Rh-positive, 339 (23.8%) Rh negative and 276 (19.3%) who did not know their Rh blood type. Receipt of Rh immune globulin in pregnancy was reported among 19.1% of peanut allergy cases and 18.7% of shellfish allergy controls (OR 0.86, 95% CI 0.51 to 1.45) (Table 1).

**Table 1:** Risk of peanut allergy vs. shellfish allergy in association with Rh immune globulin and folic acid exposure in pregnancy, as well as reduced intake of peanut containing products in pregnancy

		No. (%) with self-reported exposure		
Maternal exposure in index pregnancy		Among mothers of cases with a peanut allergy	Among mothers of controls with a shellfish allergy	Odds ratio **(95% CI)**, cases vs. controls
**Received Rh immune globulin**	No (n = 1027)	961 (73.2)	66 (61.7)	1.00 (ref)
	Yes (n = 271)	251 (19.1)	20 (18.7)	0.86 (0.51 to 1.45)
	*Unknown (n = 121)*	*100 (7.6)*	*21 (19.6)*	*--*
**Initiated a folic acid supplement before conception**	No (n = 560)	508 (38.7)	52 (48.6)	1.00 (ref)
	Yes (n = 817)	764 (58.2)	53 (49.5)	1.48 (0.99 to 2.20)
	*Unknown (n = 42)*	*40 (3.1)*	*2 (1.9)*	*--*
**Initiated a folic acid supplement after conception**	No (n = 53)	49 (9.0)	4 (7.3)	1.00 (ref)
	Yes (n = 536)	485 (88.7)	51 (92.7)	0.78 (0.27 to 2.24)
	*Unknown (n = 13)*	*13 (2.4)*	*0 (0.0)*	*--*
**Took a folic acid supplement throughout pregnancy**	No (n = 91)	87 (7.1)	4 (3.9)	1.00 (ref)
	Yes (n = 1232)	1134 (92.2)	98 (95.2)	0.53 (0.19 to 1.48)
	*Unknown (n = 10)*	*9 (0.73)*	*1 (0.97)*	*--*
**Reduced intake of peanut-containing products in pregnancy**	None (n = 1095)	1013 (77.6)	82 (76.6)	1.00 (ref)
	Somewhat (n = 236)	222 (17.0)	14 (13.1)	1.28 (0.72 to 2.30)
	Completely (n = 82)	71 (5.4)	11 (10.3)	0.53 (0.27 to 1.03)
	*Unknown (n = 58)*	*52 (3.8)*	*6 (5.3)*	*--*

Initiation of folic acid tablet supplements before or after conception, or throughout pregnancy (OR 0.53, 95% CI 0.19 to 1.48) was not associated with a significantly different risk of peanut allergy (Table 1).

Complete reduction in intake of peanut-containing products in pregnancy was associated with a non-significantly lower risk of peanut allergy among cases (5.4%) than controls (10.3%) (OR 0.53, 95% CI 0.27 to 1.03) (Table 1).

## Discussion

We evaluated three different antenatal maternal exposure factors in association with childhood peanut allergy. We did not detect an association with either Rh immune globulin or antenatal folic acid tablet supplementation and peanut allergy. Reduced ingestion of peanut products in pregnancy was associated with a non-significantly lower risk, however.

We chose to compare peanut-allergic children to those will shellfish allergy, as peanut allergy generally occurs early in life, whereas shellfish allergy often occurs later in life. We therefore reasoned that prenatal or early life factors would be more likely to affect the development of peanut as opposed to shellfish allergy.

Strengths of our study are the large number of individuals surveyed through a large national database, as well as its novelty. Study limitations include a guardian-reported diagnosis of peanut or shellfish allergy in the offspring, the unknown severity of such allergy, a recall bias about the exposure risk factors assessed, and incomplete responses to some questions. Folic acid supplementation use was so widespread among respondent mothers that it may have been difficult to detect a true effect of folic acid supplementation between cases and controls. While intentional dietary peanut consumption in pregnancy was assessed, accidental ingestion of trace amounts in food, or sensitization via transcutaneous exposure was not. In addition, we did not record the demographic features of the respondents, non-respondents and their children, which may alter the internal and external validity of our findings. The first factor studied was the administration of intravenous Rh immune globulin to pregnant women, an important immunomodulatory strategy commonly used to prevent the development of alloimmunization to fetal Rh antigens. The second factor studied was use of antenatal folic acid supplements, which has roughly paralleled the rise in allergic conditions [11]. Folate supplies methyl groups can epigenetically alter DNA and permanently affect gene expression resulting in reduced expression of Fox P3 and development of T regulatory cells [7]. This might predispose to the development of allergy, as evidenced in animal [12] and human studies [11]. Since both Rh immune globulin and folic acid have demonstrated benefits, future controlled studies would be difficult to conduct, and their absence of an association with peanut allergy may be reassuring to mothers.

The third factor studied -- reduced maternal ingestion of peanut products -- was associated with a non-significantly lower risk of peanut allergy in the offspring. The observation that some children experience allergic reactions to their first ingestion of peanut products suggests that sensitization may occur *in utero*, leading to recommendations of avoidance of peanut consumption during pregnancy and lactation [9]. However the risk of peanut allergy may be higher despite avoidance of peanut ingestion [1], possibly due to transcutaneous, rather than oral sensitization [13], a factor not addressed in this study.

Further studies are needed to delineate the risk factors that may contribute to the development of peanut allergy.

## Sources of funding

Supported by a grant-in-aid from the Division of Allergy and Clinical Immunology, St. Michael's Hospital, Toronto, Ontario, Canada

## Abbreviations

CI: confidence interval; DNA: deoxyribonucleic acid; OR: odds ratio.

## Competing interests

The authors declare that they have no competing interests.

## Authors' contributions

KB conceived of the study, participated in study design, and manuscript preparation. CL participated in study design, set up the web-based questionnaire, performed statistical analysis, and participated in manuscript preparation. JR participated in study design, data analysis, and manuscript preparation. All authors have read and approved the final manuscript.

## Appendix A

### Allergy questionnaire

• The following questions ***only ***apply to the pregnancy when you carried your **eldest **child with an allergy.

• Please answer each question as fully and accurately as possible.

• Please only provide **one **response to each question.

• You may need to check with your doctor to answer some of the questions, so take your time.

• Your responses will help us determine what factors during pregnancy may or may not relate to the development of an allergy in your child.

1. Did the mother of the current child with allergy receive Rh immune globulin (RhoGAM) during that pregnancy? (You may need to check with the person who took care of you in your pregnancy, as this information will be recorded in your prenatal chart)

Yes

No

Do not know

2. At the time that the child with allergy was conceived (at the time that you actually became pregnant) was the mother of the child with allergy taking a multivitamin supplement containing folic acid or a folic acid supplement?

Yes

No

Do not know

3. If the mother of the child was NOT taking a multivitamin supplement containing folic acid or a folic acid supplement at the time that the child was conceived, did the mother ***start ***taking a supplement one once she suspected or knew that she was pregnant?

Yes

No

Do not know

4. During the above pregnancy, which of the following best describes how much, if any, peanut or peanut containing products the mother ate during pregnancy?

I continued to eat peanut containing products without any special restrictions.

I ate fewer peanut containing products than usual, but I did not go out of my way to avoid them (for example, I did not specifically read food labels to make sure that they were free of small amounts of peanut product; OR, I know that I ate some peanut products during pregnancy, but not very often).

I was very careful to completely avoid peanut containing products during pregnancy, and, as far as I know, I did not consume any.

Other (please explain)

5. After the above pregnancy, which of the following best describes how much, if any, peanut or peanut containing products the mother ate during breastfeeding?

I continued to eat peanut containing products without any special restrictions.

I ate fewer peanut containing products than usual, but I did not go out of my way to avoid them (for example, I did not specifically read food labels to make sure that they were free of small amounts of peanut product; OR, I know that I ate some peanut products during pregnancy, but not very often).

I was very careful to completely avoid peanut containing products during pregnancy, and, as far as I know, I did not consume any.

I did not breastfeed
